# Observations upon Treatment of Yellow Fever

**Published:** 1873-08

**Authors:** Joseph Jones

**Affiliations:** Professor of Chemistry and Clinical Medicine in the Medical Department of the University of Louisiana; Visiting Physician of the Charity Hospital, New Orleans, La.


### SELECTIONS.

---

### OBSERVATIONS UPON TREATMENT OF YELLOW FEVER.

BY JOSEPH JONES, M.D.,

*Professor of Chemistry and Clinical Medicine in the Medical Department of the University of Louisiana; Visiting Physician of the Charity Hospital, New Orleans, La.*

[Reprinted from the American Practitioner for 1873.]

1. Yellow fever is a self-limited disease, and can not be arrested by drugs. The poison of yellow fever, as well as the deleterious products resulting from the chemical changes which it excites, are eliminated mainly by the skin and kidneys. Black vomit is the *result* of the action of the yellow fever poison upon the blood and upon certain organs. It should neither be regarded as the active cause, nor be treated as *the disease.* Black vomit must be viewed as a *result* and not as a *cause* of diseased action. Therefore the functions of the skin and kidneys should be promoted by suitable means during the progress of the disease. During the early stages the physician should employ those measures which are best adapted to equalize the circulation and promote the regular and free exercise of the functions of the skin and kidneys. Stimulating diuretics should, as a general rule, be avoided, as they tend to increase the irritation and congestion of the kidneys. A favorable impression may be made upon the circulation and upon the skin by the free use of the hot mustard foot-bath, by the vapor-bath, and in certain cases by the warm water-bath. The action of the skin and kidneys may be promoted by draughts of lemonade, and of warm decoctions of mild diuretics, as orange-leaf and sage tea, and water charged with carbonic acid.

2. The diet may be light but nutritious. Beef-tea, chicken-tea, corn and rice-gruel, and barley-water, are the best forms of nourishment, and should be continued at regular intervals throughout the active stages of the disease. Solid food, and even bread, should be avoided. In many cases the preceding measures, accompanied by absolute rest in bed and the careful and continuous attention of an experienced nurse, will be all that is required. Alcoholic stimulants should be used with caution, and their effects noted. They have proved beneficial in certain cases attended with great prostration in the stage of febrile excitement. Champagne, when pure, is perhaps one of the best forms of alcoholic stimulants, from the presence of the carbonic acid with which it is charged.

3. Efficient but gentle purgation in the *early part* of the *first stage* of active febrile excitement may prove beneficial in relieving in a measure the congestion of the kidneys and liver, and in removing fecal matters from the bowels. If mercurials are employed, they should be used in the early part of the first stage, not later than the second day of the disease. For an adult from eight to twelve grains of calomel or blue mass will be sufficient. Purgatives should not be administered in the second stage of calm.

4. Quinine may prove beneficial in the *earliest stage* of the disease by its effects upon the nervous system, and by its power of diminishing the temperature and equalizing the circulation; but this drug has no such curative effect in yellow fever as it has in paroxysmal malarial fever. Yellow fever will run a definite course, and pass through a definite series of changes, whether quinine be administered or withheld. After a careful examination of the statements of Blair and others, we have failed to discover any facts or cases by which the power of large doses of quinine to *abort yellow fever* can be fully and unequivocally established. It is very evident from his own statements, that the action of Blair's favorite compound of calomel and quinine, having the symbol 20x24, was very uncertain; and questions may be raised as to whether the cases said to have been aborted were yellow fever at all, or whether they may not have been some form of malarial paroxysmal fever, or whether they may not have been the milder forms of yellow fever, which would most probably have progressed regularly to convalescence after the hot stage of febrile excitement. Our own experience, as well as that of many others, has not accorded with the statement that after yellow fever has been established it *can be aborted.* Of course it would be entirely unnecessary to argue the question with those whose diagnostic powers are so acute that they are able "to detect a case of yellow fever before the supervention of the hot stage."

The power of quinine not only to arrest but also to ward off paroxysmal malarial fever is undoubted; and it has been used extensively, not only in the treatment of yellow fever, but more recently as a prophylactic. Dr. Newkirk, who was at Asuncion during the recent severe epidemic of yellow fever, assured Dr. Wm. Nathaniel Hiron, of Buenos Ayres, that the mortality was small, and that quinine was very generally and extensively used; and he expressed his belief that quinine was prophylactic, and that its continuous use in a healthy person during an epidemic caused any disease that showed itself to be mild and tractable.

Dr. Hiron, in his account of the recent severe epidemic of yellow fever in which Buenos Ayres, with a population not larger than that of New Orleans, lost, according to the most accurate estimates, nearly twenty thousand of her citizens, records the additional fact, illustrating the prophylactic properties of quinine, that "of eleven *practicantes* (dressers) of the Hospital de Hombres eight took quinine in doses of three grains daily. All of these had fever of a benign form. Three took no quinine; these had the fever very severely, and one died."

While the facts relating to this important subject are too few to warrant any decided conclusion as to the propriety and necessity of using quinine as a prophylactic by those exposed to the yellow fever atmosphere, at the same time there are facts that indicate that quinine acts not so much as an "antidote" to the poison, but as an "antidote" to the *effects* of the poison in the system, by preserving the integrity of the blood, regulating and promoting excretion, equalizing the circulation, and fortifying the nervous system against the action of the poison. According to Binz, quinine has the power of arresting putrefaction and fermentation, and is an active poison for all low organisms, animal and vegetable: and Dr. Grace Calvert has confirmed the observations of Binz, and announced the power of quinine to prevent the development of fungi.

These facts have been applied to the explanation of the effects of quinine upon the process of inflammation. Thus, according to Conheim's views, pus being mainly a collection of white blood-globules which have passed through the walls of the vessels—the alkaloid arrests, or at all events diminishes, the formation of pus during the course of inflammation. The well-established effect of quinine in producing a *decrement of temperature in fever* has been referred to its power of destroying the ozonizing power of certain substances; and as the red corpuscles have this power, quinine in the blood is supposed to diminish the oxydation of tissue, and thus to lessen the production of heat. Thus Ranke and Keener found that the tissue changes were diminished under the action of large doses of quinine. Buntz has recorded the observation that quinine, in ten-grain doses, lessens the daily excretion of urea by one-third or more; and Unruh has found the same to occur when quinine is administered in fevers. Harley added quinine to blood, and found that it took up less oxygen and gave off less carbonic acid than blood which had not been thus treated. Zuntz and Schute have employed the changes in the alkalinity of the blood for the determination of the same fact. Thus, if fresh blood be drawn, a development of acid begins in it, and continues, at first rapidly, then more slowly, till putrefaction sets in; and as this acidification depends on oxydation, the diminished alkalinity of the blood thereby produced furnishes a test of the rapidity with which oxydation proceeds; and it has been determined by the experiments of Zuntz, Scharænbroich, and Schute that quinine, bebeerine, cinchonine, and picrate of sodium lessen, in different degrees, the production of acid, and consequently prevent the oxydation of blood.

The experiments of Binz are especially important in their bearing upon the question of the direct action of quinine upon chemical changes of the blood, or of its indirect action through the nervous system, which show that when putrefying liquids are injected into the circulation, the temperature of the body rises; but if the fluids be previously mixed with quinine, whereby the putrefactive processes are arrested or destroyed, the rise in temperature is either entirely arrested or considerably diminished. Such experiments not only throw light upon the therapeutic action of such alkaloids as quinine, but they also illustrate, as it were, the very nature of the processes of those diseases, the effects of which they modify or counteract, by the peculiar chain of chemical actions which they induce in the blood.

5. While local blood-letting may be beneficial in the first stage, when practiced chiefly for the relief of local congestions of the stomach and kidneys, general blood-letting is injurious on account of its depressing effects upon the heart and nervous system. Cut cups should be employed with caution, and in the majority of cases they are unnecessary. The circulation will best be influenced by dry cups, sinapisms, and hot mustard foot-baths. Blood-letting, either in large or small quantities, repeated at intervals, is injurious, because it permanently reduces the pulse, prostrates the powers of life, and quickens the fatal termination.

6. The employment of the mineral acids internally, as the nitro-muriatic from its supposed beneficial effects upon the jaundice, as well as of the tincture of the sesquichloride of iron, from its supposed power of arresting or preventing black vomit, is of very doubtful propriety. If the view be correct that black vomit is intimately associated with and even dependent upon impairment, if not complete suppression of the functions of the kidneys, and if to a certain extent it be an effort of nature to relieve the blood of certain poisonous constituents, such agents can have little or no remedial power, and they are in many cases directly injurious by their irritant action upon the congested, irritated, and softened gastric mucous membrane.

7. While opium and its preparations may in certain cases attended with sleeplessness and great restlessness in the first stage, produce favorable results, at the same time they possess no power of arresting or curing the disease; and should be used with great caution, as they may act with great energy and even poisonous effects when the function of the kidneys is impaired or arrested. This observation applies equally whether opiates be administered by the mouth or by subcutaneous injection.

8. The maintenance, as far as possible, of absolute rest in the recumbent posture. This precaution appears to be indicated by the results of experience, as well as by the *lesions of the heart*, which I have shown by careful post-mortem examinations to be characteristic of this disease. The central organ of the circulation is structurally altered and enfeebled in yellow fever. The muscular structures of the heart present alterations similar to those observed in the liver and kidneys. Oil and granular albuminoid or fibroid matter is deposited within and around the muscular fibrillæ, and the organ, after death, presents a yellow, flabby appearance. In some cases time is required for the restoration of its free and vigorous action, and this result is impossible without absolute and continuous rest in the recumbent posture.

*Every case of yellow fever* should be regarded as *serious*, however slight the symptoms may appear; and on account of the profound structural alterations of the heart, liver, and kidneys, and the profound alterations of the blood, the closest medical attendance and the most careful nursing is demanded.

9. The maintenance of free ventilation, and at the same time the avoidance by proper coverings of sudden changes of temperature.

I have shown by numerous careful analyses of the urine, and by microscopical examinations of the kidneys after death, that in fatal cases the lesion of these organs is profound. The results of these investigations afford an explanation of the fac that sudden changes or depression of temperature often cause sudden and fatal changes in cases of yellow fever. By sudden depressions of temperature the function of the skin is diminished or arrested, internal congestions promoted and augmented in the enfeebled state of the circulatory and nervous systems which characterize the second stage of calm and depression, and the already crippled kidneys have an additional amount of work thrown upon them, while, at the same time, they are still further incapacitated for the performance of this work by the increased congestion.

10. The sudden fatal termination of many cases of yellow fever is to be referred chiefly to the sudden arrest of the function of the kidneys. Complete suppression of urine in yellow fever is of more fatal import even than black vomit, which it accompanies and precedes. In cases of suppression of urine in yellow fever the malpighian corpuscles and tubuli-urinifiri are filled with granular albuminoid matter, oil-globules, and detached epithelial cells. If the cessation of the excretion of urine was due simply to capillary congestion or defective enervation, it might be met by appropriate remedies; but the results of my chemical and microscopical examinations have placed in a clear light the reason of the impotency of all measures heretofore proposed for the relief of this fatal symptom.

The tincture of ergot has been said to have restored the excretion of urine, but this powerful agent has failed in my hands. The careful physician endeavors to promote the regular action of the kidneys, as indicated in section 1, from the very inception of the disease. As long as the kidneys excrete urine freely we may entertain hopes of recovery, even though black vomit and jaundice may have supervened.

As a general rule, suppression of the urinary excretion is speedily followed by restlessness, delirium, and coma, and in some cases convulsions. It is folly to expect any good results from sedatives and the various preparations of opium in such cases: Counter-irritants to the surface, and the prolonged use of the hot and warm baths alone promise any good.

11. Yellow fever is a self-limited disease, occurring, as a general rule, but once in a life-time. The constitution of the blood and even the textures of the body are altered. The most important organs, as the heart, kidneys, and liver, as well as the most important nutritive fluids, are profoundly impressed. These changes of the blood, heart, kidneys, and liver, and perhaps also of the nervous system, may be compared to the profound changes induced in the blood and organs, and especially in the integument, by the small-pox poison. I have shown by careful analyses that when the kidneys cease acting in yellow fever, urea and carbonate of ammonia and bile accumulate in the blood, brain, liver, and heart. Many of the nervous symptoms characteristic of yellow fever are referable to the retention of the bile and the constituents of the urine in the blood.

If this view be correct, we can not by *drugs arrest* or *cure* yellow fever any more than we can arrest or cure by drugs small-pox, measles, or scarlet fever. If drugs accomplish the effect of promoting the free and regular action of those emunctories through which the poison and the product of its action are eliminated, and if, further, they tend to preserve the integrity of the blood, and to sustain the action of the circulatory and nervous systems, they will without doubt achieve much good, and perhaps all that we are justified in looking for in the present state of our knowledge.

By judicious treatment, and by proper attention to ventilation, diet, and rest, we place the patient in that condition best adapted to the successful elimination of the poison and the products of its action, but we do not arrest or cure the disease, as we certainly may do in paroxysmal malarial fevers, by the proper administration of quinine.

